# Retrospective Evaluation of Patients Leaving against Medical Advice in aTertiary Care Teaching Hospital

**DOI:** 10.5005/jp-journals-10071-23137

**Published:** 2019-03

**Authors:** Rubina K Mahajan, Parshotam L Gautam, Gunchan Paul, Ramit Mahajan

**Affiliations:** 1-3 Department of Critical Care Medicine, Dayanand Medical College andHospital, Ludhiana, Punjab, India; 4 Department of Gastroenterology, Dayanand Medical College and Hospital, Ludhiana, Punjab, India

**Keywords:** Discharge against medical advice, Leave against medical advice

## Abstract

**Background:**

Leaving against medical advice (LAMA) is a worldwide healthcare problem, occurring due to various contributing factors, seen more commonly indeveloping countries like ours.

**Aim:**

To retrospectively study the prevalence of LAMA along with its affectingfactors.

**Methods:**

We screened the hospital record of a tertiary care teaching hospital forone year, after obtaining approval from the institutional ethicalcommittee. Patient demography, disease characteristics and status at thetime of LAMA were noted and statistically analysed.

**Results:**

During the study period, 4.95% patients took LAMA. The mean age was 47.2±21years (range newborn to 103 years) with 2:1 Male: Female ratio. Forty ninepercent of patients resided in rural areas and around 1/3rd were dependenton others for their living. The mean length of stay in hospital was 6.1±9.3days. Around 60% patients required mechanical ventilation and 51% patientshad been explained guarded prognosis. About 53% of patients taking LAMAwere admitted in medical wards, trauma being the most common diagnosis(17.2%). History of alcohol abuse and poisoning with suicidal intent wasseen in 11.47% and 3.9%, respectively.

**Conclusion:**

The number of patients taking LAMA from our country is quite high. This necessitates formulation and implementation of strategies to reduce the prevalence of LAMA discharges like further investigations to look into the causes contributing to patients taking LAMA, attending to substance abuseissues, recognizing psychological factors and strengthening the socialsystems, encouraging insurance cover, helping patients’ treatment expensesthrough charity care and optimizing healthcare delivery and patient centredpolicies.

**Key messages:**

LAMA is a global health issue precipitated by unemployment and alcohol abuse, commonly taken due to financial reasons. This necessitates a strong social system and national health insurance schemes to reduce the cost of treatment.

**How to cite this article:**

Mahajan RK, Gautam PL, *et al*. Retrospective Evaluation of Patients Leaving against Medical Advice in a Tertiary Care Teaching Hospital. IndianJ Crit Care Med 2019;23(3):139-142.

## INTRODUCTION

The patient's withdrawal of treatment before the recommendation of discharge by the treating physician is diversely abbreviated as LAMA (leaving against medical advice), DAMA (discharge against medical advice), SAMA (signing against medical advice)^1^. It is a worldwide phenomenon in the healthcare system^2,3^. The prevalence rate of LAMA in literature varies from 0.002%^4^ to around 43%^5^ in different regions and patient populations around the world.

The common reasons for taking LAMA have been documented to be hospital associated (size, location or type as in teaching or non-teaching) ^6^ or patient associated (financial constraints, socioeconomic status, substance abuse, dissatisfaction with treatment, communication gap with the physician)^7–10^ or disease associated (clinical characteristics, psychiatric illnesses, cirrhosis, HIV etc). The prevalence is higher in developing than developed countries^1,7^.

Apart from being associated with a stigma^5^, these patients often have incompletely treated medical problems or may still be severely ill at the time of self-discharge leading to higher rates of morbidity, mortality, incidence of re-admission and complications, hence contributing to excessive treatment costs^11,12^. These have also been considered as high-risk events leading to malpractice litigation^2,5,13^. Therefore, avoiding the clinical situation of LAMA will not only benefit the patients but also the health care system. Thus we planned to evaluate the record of patients who left against medical advice.

### Aims and Objectives

- To retrospectively study the prevalence of LAMA in a tertiary care hospital
- To study the demographic and patient factors affecting for LAMA

### Subjects and Methods

We conducted a retrospective study on LAMA patients in a tertiary care teaching hospital in North India. The study was approved by the institutional ethical committee (IEC). All consecutive patients who left Against Medical Advice (AMA) between 1st Jan 2015 and 31st December 2015 from the hospital were included in the study. LAMA request, as per the hospital practice, is considered when a relevant form is signed by the patient or his legal guardian. To find out the magnitude, total inpatients and LAMA patients in year 2015 were known from the record room. LAMA files were screened to see the patients’ demographic profile (age, sex, occupation, geographic area), personal history (alcohol consumption and substance abuse), diagnosis and disease characteristics and duration of hospital stay. Effect of mechanical ventilation and impact of explaining guarded prognosis to the patient's relative on incidence of LAMA was also studied.

Data was recorded, tabulated and statistically analyzed using the statistical software SPSS version 16.0 for Windows (SPSS Inc., Chicago, IL). Data from the emergency and ICU was analyzed individually and also compared. The quantitative variables are presented as mean ± SD (standard deviation) and categorical variables are summarized by absolute frequencies and percentages.

## RESULTS

During the 12-month study period, there were 65146 hospital admissions from the emergency and outpatient department. A total of 3227 (4.95%) patients took LAMA.

The demographic profile of the patients is outlined in Table 1. The age range of the patients who left AMA varied from newborn to 103 years, the mean age being 47.2±21 years. Seventy seven (2.4%) patients were infants. There were 2161 (67%) males and 1066 (33%) females with Male: Female (M:F) ratio of 2:1. Around forty nine percent of (n=1565) were from rural areas and 51.5% (n=1662) were from urban areas. Maximum number of patients belonged to the Sikh community (n=1681, 52.1%), followed by the Hindus (n=1475,45.7%), Muslims (n=69, 2.1%) and Christians (n=2, 0.1%). The study population consisted of patients from business class, service class, labourers, private job holders, but the majority of patients were unemployed and dependent (n=989, 30.6%) on others for their living. The occupation of the patients is further elaborated in Table 2 (classified according to the Kuppuswamy's socioeconomic status scale)^14^.

**Graph 1:**
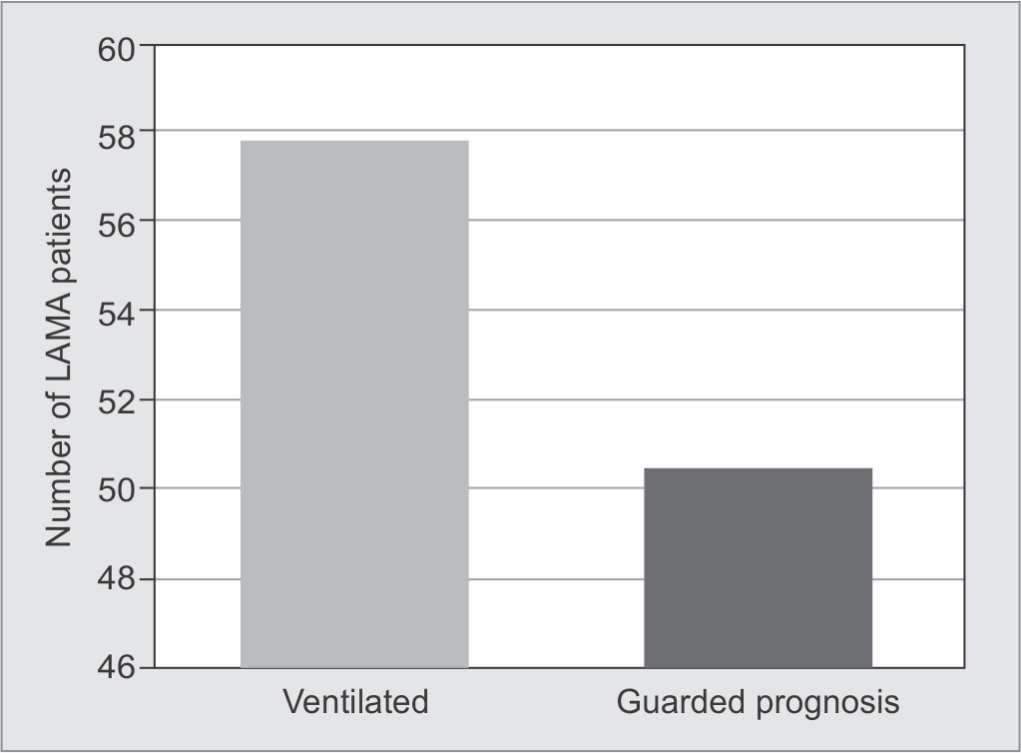
Bar diagram displaying the number of LAMA patients who were on mechanical ventilation and had been explained guarded prognosis

**Graph 2:**
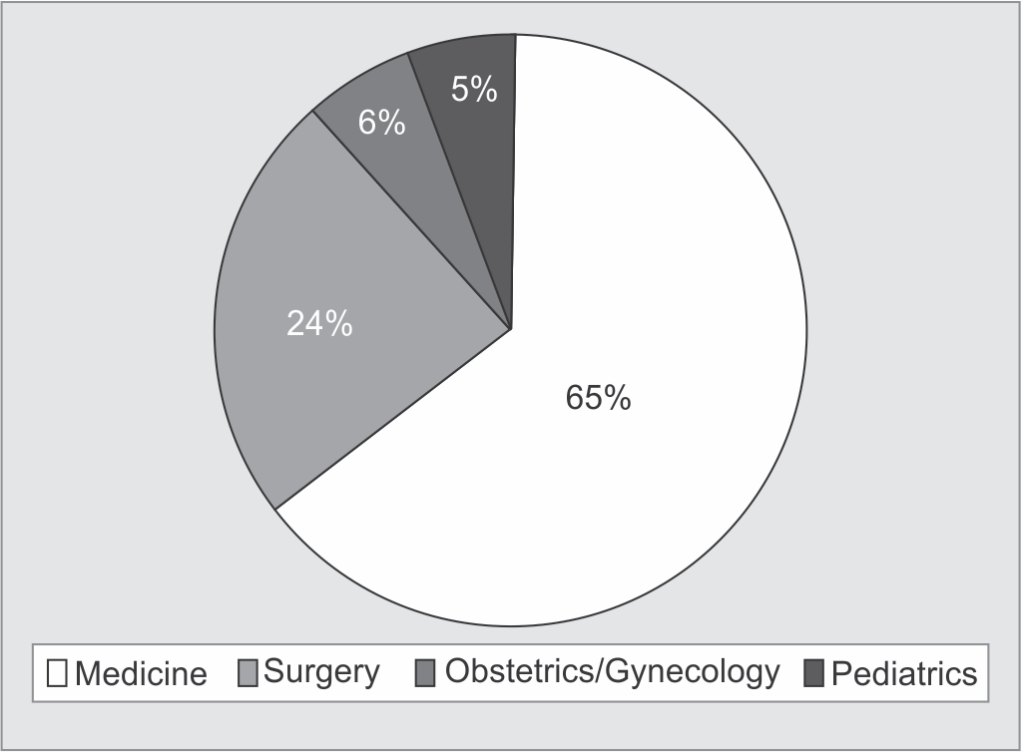
Pie chart showing the distribution of patients according to the disease speciality

There were 1866 (57.8%) patients on mechanical ventilation while in process of LAMA. Around fifty one percent (n=1632) patients had been explained a guarded prognosis by the physician (Graph 1). The mean length of stay (LOS) in the hospital was 6.1±9.3 days. Trauma was the most common diagnosis amongst the patients taking LAMA (n=555, 17.2%). Patients admitted to medical wards and ICUs contributed to more than half (n=1712, 53.05%) of the LAMA patients, followed by patients admitted in surgery (n=767, 23.8%), gynaecology (n=193, 5.9%) and paediatric wards (n=180, 5.6%) (Graph 2). Three hundred and seventy patients (11.47%) had a history of alcohol abuse and 129 (3.9%) patients were admitted with a history of poisoning with suicidal intent.

**Table 1:** Demographic data of patients

*S. No.*	*Charcteristic*	*Number (%) of DAMA patients*
1	Mean Age	47.2±21
2	Sex Male	2161 (67%)
	Female	1066 (33%)
3.	Religion Sikh	1681 (52.1%)
	Hindu	1475 (45.7%)
	Muslim	69 (2.1%)
	Christian	1 (0.1%)
4.	Urban	1662 (51.5%)
	Rural	1565 (48.5%)

**Table 2:** Demographic profile of study population

Unemployed	1226 (38%)
Unskilled	253 (7.8%)
Semiskilled	29 (0.1%)
Skilled	425 (13.2%)
Clerical/shopkeeper/farmer	761 (23.6%)
Professional	456 (14.3%)
Not told	45 (1.4%)

*According to Kuppuswamy's socioeconomic scale

## DISCUSSION

LAMA is a global issue with a wide range of prevalence depending upon the geographical region and patient type, challenging the healthcare providers and physicians because self discharge is associated with many unfavourable complications, higher readmission rate^8,9^, healthcare expenditure and professional liability^15^.

In this retrospective evaluation conducted in a tertiary care hospital of North India, we found that the overall prevalence of LAMA in the hospital was 4.95%. This is comparable to the few retrospective reports from developing countries showing LAMA prevalence rate of 1.94-13%^14^. The incidence of LAMA has been studied to be higher in developing countries like India where the healthcare system is an amalgamation of national public health system, alternative medicine practitioners and private hospitals^1,7^. Recent retrospective and prospective evaluation done on LAMA patients by our study group in tertiary care teaching hospitals of North India found the prevalence of LAMA to be 3.3% from the hospital^3^, 2.4% from the emergency and 15% from the intensive care units (ICUs)^8^. In another survey conducted in a private Indian setup, 3.8% of the patients who presented to the emergency department left against medical advice^7^.

The majority of patients in our study cohort belonged to the middle age, probably due to the economic and social pressure in this age group ^15^. As studied in literature, the age group of patients taking LAMA may vary from middle age to elderly, depending on the patients taking LAMA in that disease specific study population^8,11,16^. The higher incidence of LAMA in this prolific age group is a matter of concern as this increases the overall economic burden on the country. The M:F dominance is in conformity with the earlier studies, probably due to the fact that trauma, the most common diagnosis in our group of patients is predominant in males^17^. In our country, this could also be due to the gender related social issues as females, being the vulnerable group, are viewed as economic burdens for the family, and hence denied access to medical treatment, thus accounting for their low hospital admission rate ^7^.

LAMA in our setup was most commonly seen in patients of Sikh and Hindu religion. Probably, it is relative percentage population as Sikh and Hindu community makes most of the population in Punjab. According to the Census 2011 of India, Punjab has a population of around 27.7 million. Sikhism is the most practiced faith in Punjab, and 57.69% of the population belongs to the Sikh faith. Around 38.49% of the population practices Hinduism ^18^. The cultural differences in the rate of LAMA might be due to the reason that certain ethnicities maybe more perceptive of the healthcare system than the others^6^.

Nearly 70% of population in India stays in rural areas and belongs to the low socioeconomic class^19^. There is no consensus on whether the frequency of LAMA is more common in urban or rural setup. It all depends on the subset of population studied. Some of the studies done in patients having psychiatric illnesses have shown higher incidence in urban population in view of availability of better psychiatric hospitals in urban areas^6,20,21^. In our study, approximately half of the patients resided in the urban areas. In developing countries like India, LAMA is taken most commonly for financial reasons thus leading to a higher incidence in patients who are unemployed and dependent on others for their living^7,8^.

In the present healthcare scenario, the cost of treatment has to be borne by the family of the patient. So, the patients who were ventilated were observed to have higher chances of going LAMA, as the relatives would have anticipated a poorer outcome and high expenditure considering it a futile effort to continue further treatment. Half of patients who had left against medical advice were explained guarded prognosis. Prognostication was very physician subjective most of the times without using any definite objective criteria. Although this was not studied categorically in our study.

LAMA, in our study, was most commonly taken from patients admitted in medical wards and its allied specialities. Various studies have shown ^11,22^ that LAMA discharges are more frequently seen in medical patients because the diagnosis in these diseases usually requires more prolonged and extensive workup with repeated sampling and imaging. As far as single pathology is concerned, in concordance with other studies^7,11^, trauma was the most common diagnosis in patients taking LAMA.

We found that LAMA was more commonly seen in patients admitted with history of alcohol abuse and poisoning. The literature also suggests a consistent association of substance or alcohol abuse with decision to take LAMA. The reason for this association is not clear but the wish to acquire more drugs due to addictive behaviour has been considered as the likely reason ^2,4^. Moreover, drug abusers have a lack of trust for the health care providers, probably because of inadequate pain relief and difficulty in achieving satisfactory analgesia in opioid dependent patients for pain ^4,23^. High risk of LAMA has also been studied in adolescent patients attempting suicide, the most common mode of which was poisoning ^23^.

Our, study being retrospective in nature, had certain limitations in data collection. The reasons for taking LAMA were not documented in the patient record clearly so could not be studied. We have seen that the patients who were explained guarded prognosis had a higher incidence of LAMA but the criteria for guarded prognosis have not been defined in literature and were very physician subjective.

## CONCLUSION

The number of patients going LAMA from the hospitals in a developing country like ours is quite high. This necessitates formulation and implementation of strategies to reduce the prevalence of AMA discharges like further investigations to look into the causes contributing to patients taking LAMA, proactively attending to substance abuse issues, recognizing psychological factors contributing to LAMA, strengthening the social systems, helping patients by covering a part of their treatment expenses through charity care, helping to arrange patient transfers to less expensive community health care centers, optimizing healthcare at grassroot level by uplifting the primary and community healthcare centers and implementing national health insurance schemes along with reducing the cost of treatment in the hospitals.
